# Circular RNAs in Cancer – Lessons Learned From microRNAs

**DOI:** 10.3389/fonc.2018.00179

**Published:** 2018-05-28

**Authors:** Mihnea Dragomir, George A. Calin

**Affiliations:** ^1^Department of Experimental Therapeutics, The University of Texas MD Anderson Cancer Center, Houston, TX, United States; ^2^Research Center for Functional Genomics, Biomedicine and Translational Medicine, Iuliu Hatieganu University of Medicine and Pharmacy, Cluj-Napoca, Romania; ^3^Department of Surgery, Fundeni Clinical Hospital, Carol Davila University of Medicine and Pharmacy, Bucharest, Romania; ^4^Center for RNA Interference and Non-Coding RNAs, The University of Texas MD Anderson Cancer Center, Houston, TX, United States

**Keywords:** circular RNA, microRNA, non-coding RNA, cancer, biomarker

## Abstract

Circular RNAs (circRNA) are RNA molecules built from fragments of linear pre-messenger RNAs and other linear RNA species through a process termed “back-splicing” in which the 3′ and 5′ ends are joined together giving rise to a covalently uninterrupted loop. circRNAs are not new members of the RNA world; they were first discovered in the early 1990s. The novelty is their abundance in the mammalian cells, as recently thousands of circRNAs were discovered and annotated. The biogenesis of circRNAs is a partially characterized process, regulated by three different mechanisms: exon skipping, intron pairing, and RNA-binding proteins. On the other hand, the function of circRNAs remains largely unknown and only a handful of singular reports describe in detail the biological roles of some circular transcripts. In a very short period of time, numerous circRNAs were associated with various cancer types and were also identified in bodily fluids with the potential of being disease-specific biomarkers. In this review, we briefly describe the biogenesis and function of circRNAs and present the circular transcripts that were more than once reported in literature to be associated with cancer. Finally, we point out some of the difficulties encountered in the study of circRNAs in cancer, as we consider that taking these into account could accelerate and improve our understanding of the biologic and translational use of circRNAs in human diseases.

## Generalities about Circular RNA

Circular RNAs (circRNAs) are among the last addition to the ever-growing world of non-coding RNA (ncRNA) molecules. CircRNAs are built from the exons of linear pre-messenger RNAs (mRNAs) through a process termed “back-splicing” in which the 3′ and 5′ ends of the exon are joined together giving rise to a covalently uninterrupted loop. This classical definition of circRNA is challenged by numerous exceptions making this transcript so hard to characterize.

First, circRNAs are not new members of the ncRNA world. CircRNAs were first discovered in the 1990s when Nigro et al. observed that the exons of the tumor suppressor DCC after being spliced are joined in a different order than their genomic sequence. The upstream 5′ exons were moving downstream of 3′ exons where they were binding in a non-sequential manner, resulting in circular isoforms of DCC. They termed this novel RNA transcripts as scrambled exons (1). Two years later, it was discovered that in adult mice, the Sry gene is expressed as a circular transcript and not as a linear mRNA, which plays a crucial role in the determination of the sex during embryogenesis. The circular Sry does not bind polysomes and most probably is not translated (2). In the next decades, some anecdotal accounts describing the circularization of endogenous RNA followed (3–6), but none were convincing enough to change the perception of the scientific world regarding this topic. The breakthrough came in 2012, when Salzman et al. underlined the abundance of circRNA species in the mammalian cells, discovering and annotating thousands of circular transcripts (7).

**Figure 1:**
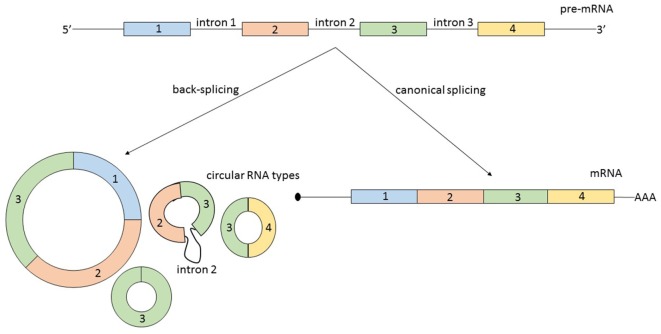
From the same linear pre-mRNA, multiple types of circular RNA (circRNAs) can be generated via back-splicing, a form of alternative splicing. CircRNAs can be composed by one or more exons and some circular transcripts are containing also intronic segments. As a generalization: circRNAs contain two or three exons, exceeding the average length, and the flanking introns are likewise longer. The relationship between the expression level of a mature messenger RNA (mRNA) and circRNA originating from the same pre-mRNA is not always correlated and is not predictable.

Second, circRNAs can originate from several types of RNA molecules. Not only exons are susceptible to circularization, but also introns, long non-coding RNAs (lncRNAs), antisense transcripts, or intergenic regions. Additionally, circRNAs originating from coding regions can be assembled from multiple exons and sometimes they also include intronic regions (8) (Figure 1). A possible generalization regarding the source of circRNA is that most of them originate from two or three pre-mRNA exons that exceed the average length and that the flanking introns are likewise unusually long (9).

Third, some circRNAs are translated into proteins (10, 11). The first circRNA which was described to associate with polysomes and to translate is circ-ZNF609 (10). Therefore, one can ask if all circRNAs are in fact ncRNA transcripts.

In conclusion, this new class of transcripts cannot be yet very precisely defined. CircRNAs can be perceived as transcripts that mainly originate from the coding region of the DNA, which occasionally prove to have a translational potential, but usually are non-coding.

## Biogenesis of circRNA

The unanswered questions regarding circRNA biogenesis does not concern the mechanism which is mostly explained, but the factors that regulate the formation of the circRNA loop. Very intriguing, circRNAs are expressed differently in various cell types of an organism and during the stages of ontogenesis, but from a phylogenetic point of view, a similar expression level between the same cell types is conserved (12). Hence, the synthesis of circRNAs seems to be a regulated process. Additionally, the relationship between the expression of an mRNA and circRNA originating from the same pre-mRNA is not linear and is not predictable. There are several publications revealing that some circRNAs are more abundantly expressed than their associated linear mRNA isoform (8, 13). Therefore, circRNAs are most probably not transcriptional noise and most likely have a biological function.

**Figure 2:**
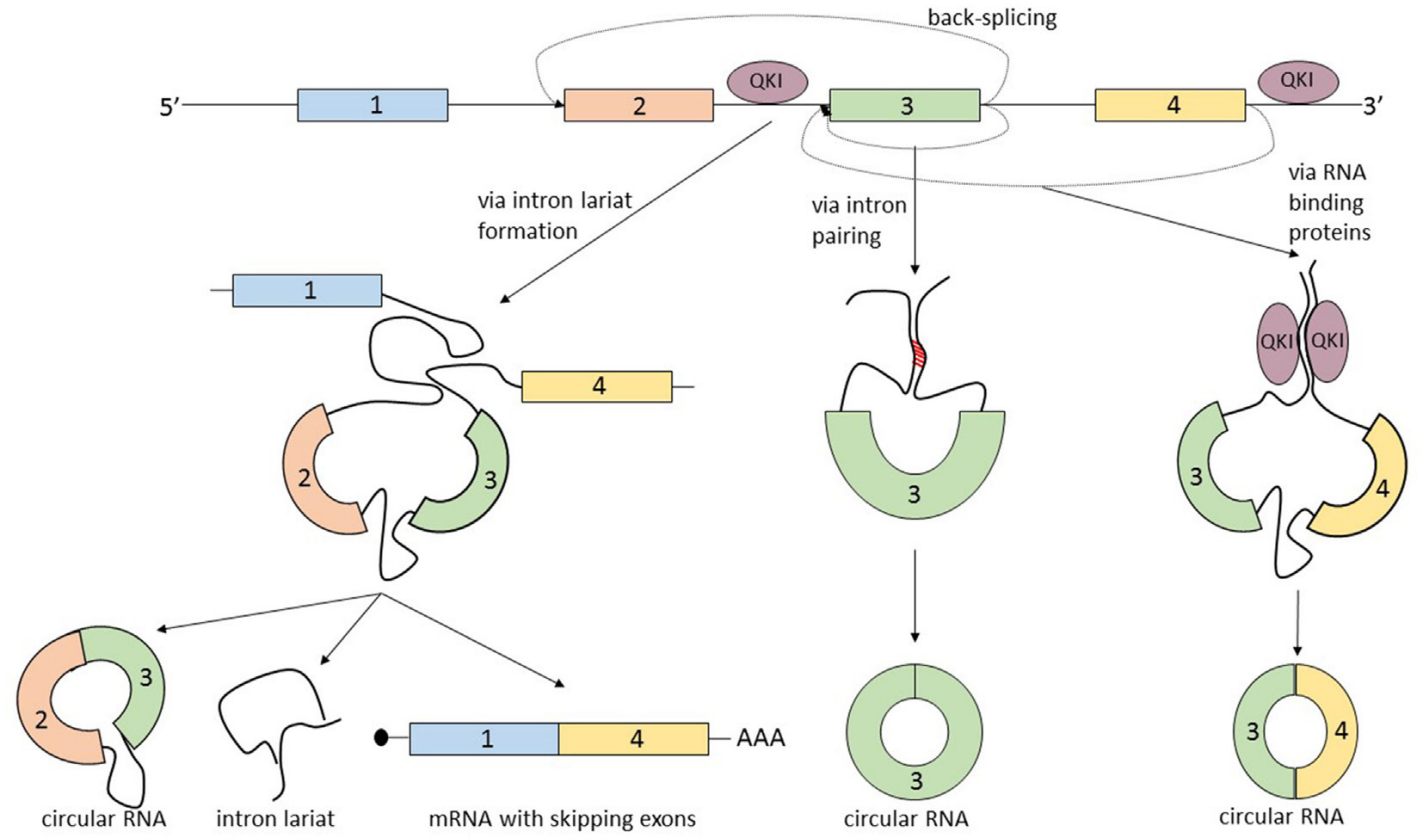
The biogenesis of circular RNAs (circRNAs) is summarized by the term “back-splicing.” Until now, three different mechanisms that promote this process were described. The first mechanism is characterized by exon skipping and intron lariat formation. In order to bring distant exons together (jumping exons), the introns build a circular lariat. The lariat formation makes the circularization of the “skipped” exons possible. This process leads to the formation of three different types of RNA molecules: circRNA, intron lariat, and mRNA with skipping exons. In the second mechanism, the circularization of the exon/exons is promoted by the complementary pairing of the flanking introns. A common hallmark of the introns that are prone to pair is the inverted ALU repeats. The third mechanism is controlled by RNA-binding proteins, which bind the neighboring introns of the future circular exon and dimerize, creating an RNA loop. The most studied proteins able to induce circularization are Quaking (QKI) and Muscleblind (MBL).

The term that summarizes the circRNA biogenesis is back-splicing, in which the head-to-tail splice junctions are joined together and form a circular transcript. But what makes this non-canonical splicing possible? There are three known processes that can lead to back-splicing (Figure 2).

The first mechanism that promotes back-splicing is exon skipping. A pre-mRNA is spliced and two transcripts are generated: a mRNA from which one or more exons are missing, and a lariat containing the skipped exons which makes the circularization possible. The exon lariat is one more time spliced (process termed intralariat splicing) and two other molecules are produced: a circRNA and an intron lariat (4). In order to confirm this mechanism, Jeck et al. researched the association of circRNAs and exon skipping transcripts. For 45% of tested circRNAs, they identified a corresponding exon skipping mRNA (9). Furthermore, these observations are supported by another group which found a linear correlation between circularization and the exon skipping phenomenon (14).

A second mechanism that leads to the circularization of exons is intron pairing: the two introns that flank the exon/exons of the nascent circRNA have a complementary structure able to bind to each other. The pairing of the flanking introns brings the splice sites close to each other creating a secondary structure that makes back-splicing possible (15–18). Computational analysis revealed that a common feature of this flanking introns are the inverted ALU repeats (9). This feature of circRNA makes it possible to predict the sites of circularization by using bioinformatic methods.

The third mechanism of circRNA formation is directed by RNA-binding proteins (RBP). The simplest example of this model of biogenesis is the interaction between the RBP Quaking (QKI) and the flanking introns of the forming circRNA. QKI proteins bind to each of the flanking introns and dimerize creating a bridge between them. This process leads to the formation of a closed RNA loop allowing back-splicing. This mechanism was demonstrated in the case of the SMARCA5 gene, where only in the presence of QKI, a circular transcript can be generated (19). A similar mechanism was described by Ashwal-Fluss et al., which showed that the biosynthesis of the circRNA, circMbl, is controlled by the Muscleblind protein (MBL). High levels of MBL bind to its own pre-mRNA and determine the back-splicing of it, leading to the inhibition of the canonical splicing and decreasing the levels of MBL, and increasing the levels of circMBL. The authors describe the circRNA biogenesis as a process that competes with canonical splicing, and as a post-transcriptional regulator of protein synthesis (20). It is not yet clear how broad the RBP mechanism of generating circRNA is and in which way it can be used to create computational predictions regarding the exons prone to circularization. There is an additional mechanism that describes the biogenesis of circRNA which actually combines two of the previous described mechanisms: intron pairing and RBP. The RBP ADAR is responsible for A-to-I RNA editing and was recently linked to the circRNA biogenesis. Because it was well known that ADAR interacts with double strand ALU repeats (21, 22), Ivanov et al. hypothesized that the ADAR enzyme by decreasing the complementary of the inverted ALU repeats of introns can lead to a downregulation of the synthesis of circRNA. These observations were confirmed by knockingdown ADAR *in vitro*. The authors detected a two-fold upregulation of 84 circRNA and the downregulation of the corresponding linear transcripts (23).

Most probably the biogenesis of circRNA is not yet fully deciphered and the above described mechanisms represent only a limited view. Ebbesen et al. consider that the three mechanisms of circRNA biogenesis can be overlapping processes and what we currently know are three perspectives on the same phenomenon (24). Future research is needed, because our understanding of the biogenesis of circRNA will enable the development of computational tools that can predict the genomic sights susceptible to circularization.

## Function of circRNA

Research regarding the function of circRNAs is still limited and challenging. Conservation of a transcript between species is always a powerful argument that supports the functionality of a molecule. Even the conservation of circRNA is disputed. The first study that affirms the conservation of circRNA mentions that there are hundreds of analogous transcripts in the brain of mouse and humans but no details exist about the similarity of the primary structure of the molecules (7). Another study that compares the neuronal circRNAs in the brain of mice and humans (separated by about 80 million years of evolution) reports that 4,522 circRNAs out of 15,849 are highly conserved between the two species (12). By comparing the number of human genes that produce circRNAs to murine genes that code circular loops, it was detected an overlap of 22%, but only 69 circular molecules share the same start and stop points (9). Guo et al. compared the same species and observed that if a mouse gene can code for a circular transcript, then in 66% of the cases the ortholog human gene can also code circRNAs. Of these genes, only one-third shared the same splicing sites for circRNA in humans and mice. This discovery led to the conclusion that the pre-mRNA fragments that give rise to circRNAs do not have a higher degree of conservation than their neighboring exons (25). Additionally, another research group studied the conservation of circRNA from the brain of pig and mice and discovered that the splice site of circularized exon loops matches between the two species at a proportion of 20.4% (26). The results between studies are divergent and the cause is easily identifiable: the conservation is higher if comparing the expression of circRNA between the same organs or tissues (i.e., brain), and if comparing the conservation of highly expressed circRNAs.

A second argument for the functionality of circRNAs is that these transcripts are expressed in a cell-specific manner and have a determined subcellular localization. Regarding their abundance, circRNAs have the highest level in brain and this feature is highly conserved (12, 26–28). In regard to their diversity, Maass et al. analyzed the expression of circRNAs in 20 different human tissues and discovered 5,225 transcripts, with the highest expression of circRNAs in platelets (3,324 circRNAs, out of which 2,339 unique to this cell type) (8). Recently, circRNAs were discovered also in bodily fluids with the potential of being disease-related biomarkers. The first account of circRNAs in the extracellular environment described over 400 different circRNAs in the saliva of healthy donors (29). Subsequently, circRNAs were detected in whole blood; over 2,400 circRNAs were discovered, which had expression levels similar to neuronal tissues (30). The world of circulating circRNAs was further characterized: circRNAs are abundant in exosomes and can be used to diagnose colorectal cancer (CRC). Compared to healthy controls, exosomes from CRC patients contain 257 new species of circRNAs, while other 67 circRNAs are missing (31). It appears that a precise mechanism controls the sorting and exporting of circRNAs into circulation via exosomes exists and needs to be revealed.

A third indirect argument of the functionality of circRNAs is their high stability compared to other RNA species. It was reported that a circRNA has a half-life of approximately 48 h (9), probably due to the resistance to RNA exonuclease. The half-life of a circRNA is two to four times longer than a mRNA. This difference in kinetics makes it difficult to interpret the relationship between the two transcripts (circRNA and corresponding mRNA) that originate from the same pre-mRNA.

**Figure 3:**
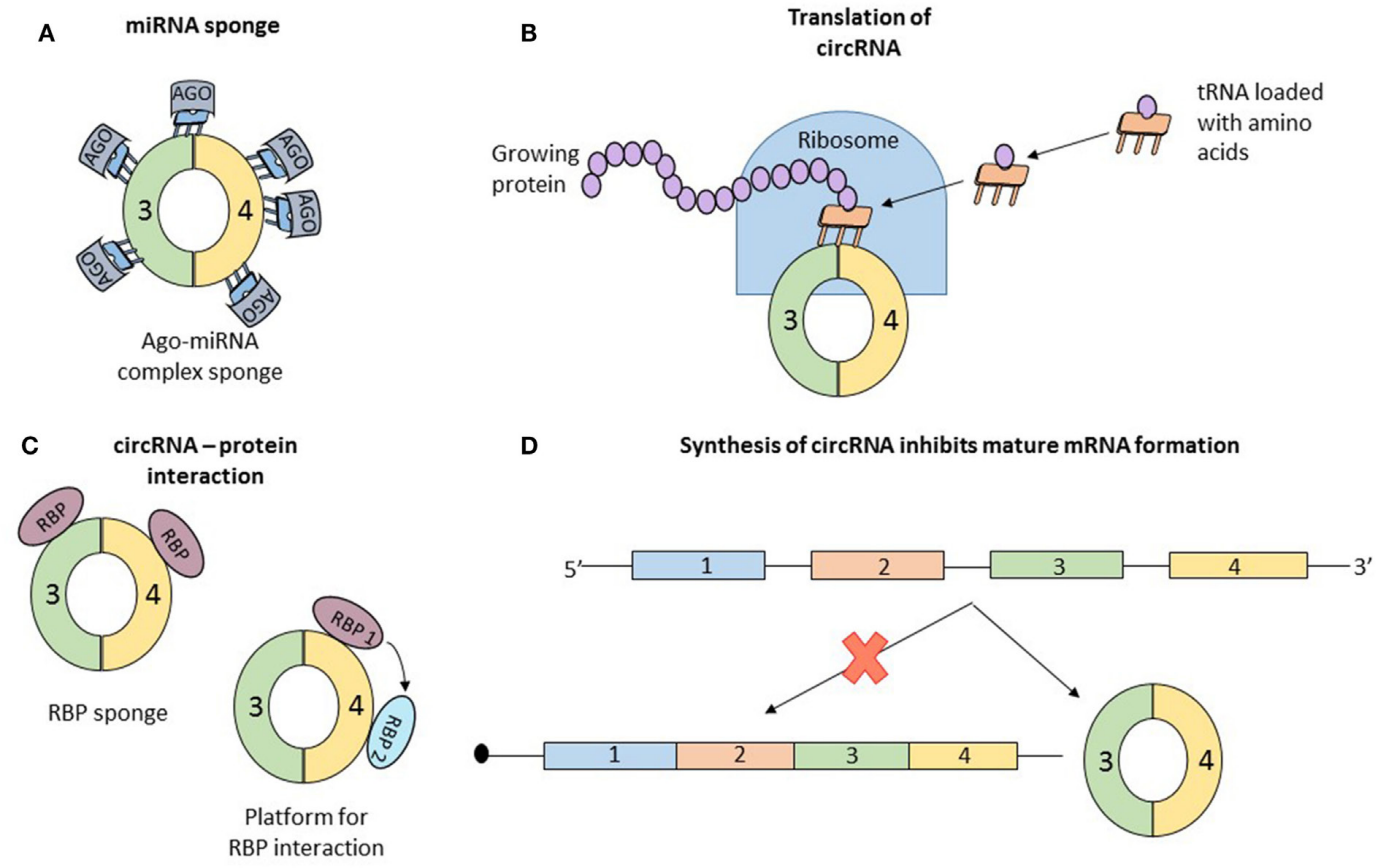
Data about the function of circular RNAs (circRNAs) is limited; until now, only four roles of circRNAs were studied in detail, and the observations regarding the function cannot be generalized to all circular transcripts. **(A)** The most studied function of circRNAs is microRNA (miRNA) sponging. By sequestering miRNA molecules that downregulate mRNAs, circRNAs indirectly increase the expression of mRNAs. Initially proposed as potent miRNA sponges which are able to bind to dozens of short RNAs, circRNAs are considered now to have the same number of interaction sites with miRNAs as other molecules. **(B)** Recently, two papers reported that circRNAs can associate with ribosomes and be translated into short proteins, long enough to contain a functional domain. **(C)** Similar to miRNA sponging, circRNAs also bind to proteins. The exact function of this interaction is not clear and numerous speculations exist: circRNA are a transport vehicle for proteins; circRNAs are sequestering proteins; circRNAs are a platform for protein interactions; or circRNAs bind to proteins and induce allosteric changes, regulating their function. **(D)** Finally, simply the formation of circRNAs can be perceived as a function of circular transcripts. It was reported that synthesis of a circRNA from a pre-mRNA competes with the formation of a linear, functional mRNA. Therefore, circRNA formation is another form of post-translational regulation.

Concrete information of the function of circRNA is scarce. Data about the mechanism of some individual circRNAs are available. But in all cases, the properties that confer functional potential cannot be extrapolated to other circRNAs. Simultaneously, two articles reported that the circRNA, antisense to cerebellar degeneration-related protein 1 transcript (CDR1as) has more than 70 binding sites for miR-7. MiR-7, associated with Argonaute proteins, binds to CDR1as, but the RISC complex does not degrade the circRNA (32, 33). CDR1as, a circRNA abundant in the brain of mammals (34), seems to act as a sponge for miR-7, which in the presence of CDR1as is strongly suppressed (Figure 3A). *In vivo*, overexpression of CDR1as leads to a phenotype characterized by impaired midbrain development, comparable to miR-7 knockdown models (32). Similarly, SRY, one of the first described circRNAs (2), has 16 binding sites for miR-38, also appearing to be an endogenous sponge (33). In order to check if other circRNAs also have sponging potential, Guo et al analyzed the interaction of 7,112 circRNAs with 87 microRNA (miRNA) families. The authors concluded that CDR1as which contains 71 binding sites for miR-7 is an exception and the next best miRNA sponge is circRNA ZNF91 which has 24 binding sites for miR-23 (25).

Recently, two papers proved that circRNAs can be translated into proteins (Figure 3B). Circ-ZNF609 has the ability to associate with polysomes and because of its primary structure containing “start” and “stop” codons, this transcript is translated in a cap-independent manner (10). One of the papers also confirmed that several circRNAs, *in vitro* and *in vivo*, associate with translating ribosomes and produce small proteins in a cap-independent way. Furthermore, the authors present the necessary characteristics of a circRNA to be translated: frequently contain the start codon of the host gene, are longer than other circRNAs, and contain a stop codon highly conserved and located near the splice site. The translated proteins originating from circular transcripts are long enough and contain a full protein domain, suggesting functional potential (11). The activity of the proteins encoded by these circRNAs is unknown and remains to be elucidated.

Several research papers report that circRNAs associate with RBP (Figure 3C). Maybe the best known example is that of circMBL. The mature MBL protein not only binds to the introns flanking the circularized exon but also interacts directly with the exons. The interaction with the exon does not affect the biogenesis of circRNA and the authors assume that circMBL can sponge excessive MBL and play a role in subcellular delivery of the mature protein (20). In another paper, it was proven that circRNAs form cytoplasmic complexes with proteins. 12 circRNAs were tested, and all of them can build complexes with proteins. A more detailed analysis using RNA-seq and immunoprecipitation showed that the RBP, insulin-like growth factor 2-binding protein 3 (IGF2BP3) can interact with three distinct circRNAs (CDYL, NFATC3, and ANKRD17) (35). These functional details open more questions than provide answers: (1) Why do circRNAs bind to proteins? (2) Are all circRNAs able to interact with RBP? and (3) What characteristics should a circRNA have to bind to a specific type of protein? Hentze and Preiss provide some hypothetical answers for these questions but experimental confirmation is still missing. The two authors propose multiple scenarios: (a) circRNAs are vehicles for RNP, delivering these molecules in a specific subcellular localization; (b) similar to the circRNA–miRNA interaction, circRNAs can be sponges for RNP, sequestering the proteins which will be unable to perform their functions; (c) circRNAs can be a platform for multiple RBPs, which can interact with each other by binding to a circularized transcript; and (d) circRNAs can bind RBPs and induce allosteric changes, regulating the function of the proteins (36). Like in the case of circRNAs acting as miRNA sponge also the hypothesis that circRNAs can interact with RBPs is disputed. You et al. predicted the capacity of circRNA to bind RBP and concluded that circular transcripts possess a lower affinity to bind RBPs than coding regions and 3′UTR of linear transcripts (28). Future research is necessary to clarify the role of the interaction of circRNAs with RBPs.

Finally, the circRNAs may not have a function, but simply the assembly of the circular transcripts can be perceived as a translational brake, a different type of post-transcriptional regulation (Figure 3D). Every time a circRNA is synthesized, the formation of the mature linear mRNA is impeded, the two transcripts (mRNA and circRNA) compete for their production. The already well-known example of muscleblind gene is a model of this phenomenon. Conditioned by the presence of the mature MBL protein, the pre-mRNA of MBL can be spliced canonical or back-spliced. If the MBL protein is abundant, it binds to the flanking introns of exon 2 of the pre-mRNA and induces its circularization generating a circRNA; if the expression of MBL is low, canonical splicing takes place and a mature mRNA is synthesized, which will be transcribed into MBL proteins. This process is highly conserved, from flies to humans, and can be perceived like an auto-feedback mechanism that controls the expression level of MBL. Exogenous MBL overexpression leads to an increased circularization of the second exon, whereas a low level of MBL induces canonical splicing (20). But once again, this process remains singular and may be an exception.

## Cancer and circRNA

The same enthusiasm observed after the discovery of the association of miRNA with cancer (37) is happening now with circRNA. In a very short period of time, numerous circRNAs were associated with various solid and hematologic malignancies. Complete reviews of the circRNAs down-/upregulated in cancers can be found elsewhere (38–40). We included in this review only circRNAs that were associated with the same type of cancer/different types of cancer in at least two publications (Table 1). Only a negligible number of circRNAs fulfill these conditions. By including circRNAs that were linked with multiple types of cancer, we observe a lack of specificity of a circRNA for a given type of malignancy.

**Table 1:** CircRNAs that were reported by two or more studies to be associated with the same/different types of cancers.

CircRNA alias(circRNA)	Host gene	Up/down	Tumor type	Function and phenotype	Reference
Hsa_circ_0022383 (hsa_circRNA_100833)	FADS2	Down	BCC	Potentially anti-tumorigenic role, by sponging different miRNAs	(42)

		Down	CSCC		(41)

Hsa_circ_0022392 (hsa_circRNA_100834)	FDAS2	Down	BCC		(42)

		Down	CSCC		(41)

hsa_circ_0001946 (CDR1as/ciRS-7)	CDR1	Up	HCC	Potentially pro-tumorigenic role, by sponging miR-7. Knockdown of CDR1as inhibits cell proliferation and invasion	(44)

		No change	HCC	Uncertain role, potential miR-7 sponge	(45)

		Up	CRC	Potentially pro-tumorigenic role, miR-7 sponge, positively regulating EGFR and IGF-1R. Knockdown of CDR1as suppresses cell invasion and proliferation	(46)

		Up	CRC	Potentially pro-tumorigenic, miR-7 sponge, leading to the activation EGFR/RAF1/MAPK pathway. *In vitro* and *in vivo* CDR1as overexpression increases the oncogenic phenotype	(47)

		Down	Glioma	Potentially anti-tumorigenic role, CDR1as is a target of miR-671-5p, and overexpressing miR-671-5p leads to downregulation of CDR1as, increasing cellular migration and proliferation	(48)

(Cir-ITCH)	ITCH	Down	ESCC	Potentially anti-tumorigenic role, by sponging miR-7 positively regulates ITCH, an inhibitor of WNT/beta-catenin. Overexpression of circ-ITCH leads *in vitro* and *in vivo* to a less aggressive phenotype	(49)

		Down	CRC		(50)

		Down	Lung cancer		(51)

		Down	HCC	Contains multiple SNPs which can modify the susceptibility to HCC	(52)

		Down	Bladder cancer	Potentially anti-tumorigenic role, by sponging miR-17 and miR-224 positively regulates p21 and PTEN. Overexpressing circ-ITCH *in vivo* leads to inhibition of metastasis	(53)

(Circ-Foxo3)	FOXO3	Down	*In vitro* and *in vivo* breast cancer models	Potentially anti-tumorigenic role, increases the expression of its corresponding transcript, Foxo3 by sponging several miRNAs. Circ-Foxo3 has an inhibitory effect on tumor growth *in vivo*	(54)

		Down	*In vitro* breast cancer models	Potentially anti-tumorigenic, builds a ternary complex with CDF2 and p21 which inhibits cell cycle progression, decreasing cell proliferation	(55)

		Down	Breast	Potentially anti-tumorigenic, blocks the interaction of MDM2 with Foxo3 and decreases the degradation of this protein, inducing cell apoptosis	(56)

Hsa_circ_0001649	SHPRH	Down	HCC	Potentially anti-tumorigenic, the low expression correlates with the tumor size and occurrence of tumor embolus	(57)

		Down	GC^a^	Potentially anti-tumorigenic, correlates with the histopathological differentiation level	(58)

		Down	CCA	Potentially anti-tumorigenic, inhibits proliferation, invasion, migration and apoptosis	(59)

		Down	CRC^a^	Potentially anti-tumorigenic, correlates with the histopathological differentiation level	(60)

Hsa_circ_0000284 (circHIPK3/bladder cancer-related circular RNA-2)	HIPK3	Up	HCC	Potentially pro-tumorigenic role, by sponging miR-124, it leads to increased cell proliferation	(61)

		Up	HCC	Possible pro-tumorigenic potential, by sponging miR-124, circHIPK3 indirectly induces proliferation and migration	(62)

		Down	Bladder cancer	Potentially anti-tumorigenic role, by sponging miR-558 circHIPK3 inhibits cancer growth and metastasis *in vivo*	(63)

		Down	Bladder cancer	Possible anti-tumorigenic role by sponging miR-124. High levels of circHIPK3 associate with better survival	(64)

(Hsa_circ_001569)	ABCC1	Up	CRC	Potentially pro-tumorigenic role, by sponging miR-145 it induces proliferation and invasion	(65)

		Up	HCC	Potentially pro-tumorigenic role correlates with TNM stage and differentiation grade. *In vitro* and *in vivo* silencing of the circRNA suppresses tumor growth	(66)

Hsa_circ_0002768 (CircMYLK)	MYLK	Up	Bladder cancer	Potentially pro-tumorigenic role by sponging miR-29a (*in silico* prediction)	(67)

		Up	Bladder cancer	Potentially pro-tumorigenic role, by sponging miR-29. Up-regulation of the circRNA induces epithelial-mesenchymal transition *in vitro* and promotes metastasis *in vivo*	(68)

(CircPVT1)	PVT1	Up	GC	Potentially pro-tumorigenic role, by sponging miR-125 family. *In vitro* silencing of circPVT1 inhibits tumor cell proliferation	(69)

		Up	AML	Potentially pro-tumorigenic role and associates with 8q24 chromosome amplicons	(70)

		Up	HNSCC	Potentially pro-tumorigenic by sponging miR-497-5p. Mutated p53 enhances the expression of circPVT1 and increases cell proliferation, migration and colony formation	(71)

hsa_circ_0075825	LINC00340	Up	BCC	Potentially pro-tumorigenic role	(42)

		Up	GC^a^	Potentially pro-tumorigenic role	(72)

circRNA_100269	LPHN2	Down	GC	Potentially anti-tumorigenic role, possible predictive tool for early recurrence after surgery	(73)

		Down	GC	Potentially anti-tumorigenic role, by sponging miR-630 inhibits tumor growth *in vitro*	(74)

*We adopted the circBase name (circRNA alias) and other additional names used by researchers for these transcripts. ^a^circRNAs that were found deregulated in serum/plasma—potential non-invasive biomarker, all other were detected in tumor tissues vs normal*.

Circular RNAs were linked to dermatologic malignancies. By performing microarray and confirming the data by qRT-PCR, Sand et al. discovered 143 up- and 179 downregulated circular transcripts in tissue samples of cutaneous squamous cell carcinoma (CSCC) compared to controls. The top 2 up- and downregulated circRNAs were hsa_circ_0070933, hsa_circ_0070934 and hsa_circ_0022392, hsa_circ_0022383, respectively (41). By performing the same analysis, the same research group reported that the top two downregulated circRNAs in CSCC have also a lower expression level in basal cell carcinoma (BCC) compared to normal tissue (42). To our knowledge, only one circRNA was linked to melanoma, the circular isoform of the lncRNA ANRIL, circANRIL. It was observed that numerous isoforms of circANRIL are present in the cytoplasm of melanoma cell lines, while the linear lncANRIL is abundant in the nucleus (43).

Most of the cancer related circRNAs reported by now are associated with malignancies of the gastrointestinal tract. Yu et al. detected CDR1as to be upregulated in hepatocellular carcinoma (HCC) compared to normal adjacent tissue and that the expression of miR-7 anticorrelates with that of CDR1as. *In vitro* experiments showed that CDR1as is an oncogene by sponging miR-7 and increasing cancer cell proliferation and invasion potential (44). More recently, another group analyzed the level of CDR1as in HCC tissue versus paired neighboring normal tissue and observed that the circular transcript has the tendency for a lower expression in malignant tissue. Curiously, a high expression of CDR1as was associated with microvascular invasion and acted as a miR-7 sponge (45). The two publications contradict to some extent one another; thus, supplementary research is needed to elucidate the real function of CDR1as in HCC, if any function exists. Furthermore, other two publications reported that CDR1as is upregulated in CRC tissue compared to paired healthy mucosa, and also both papers show independently that a high level of this circular transcript is associated with poor overall survival. Mechanistically, the overexpressed CDRas1 sponges the tumor suppressor miR-7 and the downstream target of miR-7 – EGFR – is upregulated (46, 47). CDR1as was also associated with glioblastoma, since tumor biopsies had a lower level of CDR1as compared to biopsies from normal controls. The expression of CDR1as negatively correlates with that of miR-671-5p in glioblastoma. Because this miRNA can degrade CDR1as, it can be hypothesized that miR-671-5p directly controls the expression of a circular transcript in cancer (48).

Cir-ITCH is another well-known circRNA already linked with five types of cancer. Three papers reported that cir-ITCH is downregulated in esophagus squamous cell carcinoma (ESCC), CRC, and lung cancer. The proposed mechanism of action was similar in all three articles: low levels of cir-ITCH are not able to sponge miR-7 and miR-214 which will target ITCH, an inhibitor of WNT/beta-catenin pathway. Cir-ITCH positively regulates the level of its host gene (49–51). In HCC, cir-ITCH seems similarly to be downregulated and low cir-ITCH is an independent prognostic factor. Moreover, cir-ITCH displays six different single-nucleotide polymorphism (SNPs) regions which can change the susceptibility to HCC (52). Recently, low cir-ITCH was associated with bladder cancer, and cir-ITCH plays a tumor suppressing role by directly binding miR-17 and miR-224, which if sponged lead to the inhibition of PTEN and p21 (53).

By using *in vitro* and *in vivo* models of breast cancer it has been shown that the circ-Foxo3 has a tumor suppressor potential. The authors demonstrated that high levels of circ-Foxo3 and Foxo3 pseudogene can sponge multiple miRNAs that target the mRNA Foxo3, which is transcribed in a protein with anti-neoplastic effect (54). The same study group additionally showed that circ-Foxo3 has a tumor suppressor function, independently of controlling the expression level of its host gene. Circ-Foxo3 can build together with p21 and CDK2, a complex that blocks the cell cycle progression by inhibiting the interaction of CDK2 with cyclin A and cyclin E (55). In a third paper, the authors confirmed that circ-Foxo3 is downregulated in breast tumor samples compared to benign mammary lesions. Circ-Foxo3 is additionally able to block the interaction of MDM2 with Foxo3 and rise the expression level of Foxo3, increasing the apoptosis of tumor cells (56).

Hsa_circ_0001649 is a circular transcript linked to multiple types of gastrointestinal tract cancers. In HCC, it was shown that the expression of circ_0001649 is downregulated in tumor tissue compared to paired healthy tissue (57). In gastric cancer (GC), hsa_circ_0001649 is downregulated in tumor tissue, consequently leading to expression drops in serum of GC patients after surgery, being a potential recurrence biomarker (58). The same circRNA is downregulated in cholangiocarcinoma (CCA) tissue and regulates cell proliferation, apoptosis, migration, and invasion (59). Ultimately, this circRNA is downregulated in CRC tumor tissue and in serum of CRC patients (60). Very curiously, no *in vitro* or *in silico* data exists regarding the molecular mechanism of hsa_circ_0001649, a transcript that seems to be implicated in multiple cancer types.

CircHIPK3 is the circular transcript formed from the second exon of HIPK3, a tumor suppressor gene with protein kinase activity. In HCC, circHIPK3 is overexpressed and sponges 9 different miRNAs displaying 18 miRNA interacting binding sites. Knockdown of circHIPK3, but not of HIPK3, leads to an inhibition of cellular proliferation, proving that the function of the circRNA is independent from that of the linear transcript (61). In a more recent study, it was confirmed that circHIPK3 is upregulated in HCC tissue compared to normal tissue and the level of the circular transcript anticorrelates with miR-124. The sponging of miR-124 by circHIPK3 leads to the overexpression of aquaporin 3, a transmembrane channel with tumorigenic function (62). On the contrary, circHIPK3 was reported to be downregulated in bladder cancer tissue compared to normal adjacent epithelium. Mechanistically, a low expression level of circHIPK3 cannot efficiently sponge miR-558, a miRNA which promotes angiogenesis by targeting heparanase. *In vivo* overexpression of the circRNA inhibits cell growth and lung metastasis (63). Furthermore, in a fourth study, it has been demonstrated that low levels of circHIPK3 are associated with low rate of progression free survival in bladder cancer, confirming the results of a previous research group (64). Hence, it is possible to hypothesize that circHIPK3 plays opposite roles in HCC and bladder cancer, being an oncogene and a tumor suppressor gene, respectively.

CircRNA_001569 was identified as an oncogene in CRC and HCC. In CRC, Xie et al. found that circ_001569 is upregulated and directly correlates to the T stage, N stage, M stage, and histopathological differentiation grade. The authors demonstrated that the circRNA regulates the proliferation and invasion of neoplastic cells by sponging miR-145 – a miRNA that downregulates multiple oncogenes: E2F5, BAG4, and FMNL2 (65). In HCC, the same circ_001569 is overexpressed and also correlates with the TNM stage and tumor differentiation grade, but data regarding the function of the circRNA were not provided (66).

CircMYLK is an additional circular transcript linked to bladder cancer. Huang et al. discovered circMYLK overexpressed in bladder cancer, and by using *in silico* methods, predicted that this circRNA with the lncRNA H19 could sponge miR-29a-3p leading to the overexpression of DNMT3B, VEGFA, and ITGB1 (67). In a subsequent study, the same research group confirmed their *in silico* data: circMYLK is overexpressed in bladder cancer and the expression level correlates with progression level. Additionally, circMYLK can directly bind to miR-29a-3p and control the expression level of VEGFA (68).

CircPVT1, due to the amplification of its DNA locus, is overexpressed in GC. The expression of circPVT1 and its equivalent not circularized RNA, PVT1, are inversely correlated. The association of high circular transcript and low lncRNA is an accurate overall survival prognosis marker in GC. The function of circPVT1 in GC is described only partially: this circRNA sponges the members of the tumor suppressor miR-125 family (69). Afterward, it was discovered that the same amplification of the 8q24 region containing the PVT1 gene leads to the overexpression of circPVT1 and in a lesser amount of the lncRNA PVT1 in acute myeloid leukemia (AML) patients with cytogenetic abnormalities. The phenotype of 8q24 amplification in AML is known, but remains unclear how circPVT1 contributes to this genomic alteration (70). A third article confirms the oncogenic potential of circPVT1: the circularized transcript is high in head and neck squamous cell carcinoma (HNSCC), especially in patients with TP53 mutations, and has the ability to sponge miR-497-5p (71).

One of the top overexpressed circRNAs in BCC (42), has_circ_0075825, was also found by RT-droplet digital PCR to have a high expression level in the plasma of GC patients (72).

In stage III GC, it was observed that 46 different circRNAs are deregulated, the top downregulated circular transcript being circRNA_100269 (73). In a subsequent paper from the same research group, it was confirmed that circRNA_100269 and its corresponding mRNA, LPHN2 are downregulated in GC. CircRNA_100269 has an anti-neoplastic activity by directly targeting miR-630, but further details are still lacking regarding the downstream targets of miR-630 (74).

## Understanding circRNAs Lessons Learned from the Study of microRNAs

Shortly after the discovery of miRNAs implication in cancer, these transcripts were divided in oncomiRs and tumor suppressor miRNAs. Later, a more in depth research made it clear that a miRNA, which has the ability to target hundreds of different mRNAs, is too versatile to be classified as “good” or “bad.” The role of a miRNA in tumorigenesis is largely context dependent (i.e., depending on genomic features of the tumor). The same miRNA can be an oncogene in some tumor types and a tumor suppressor gene in other cancers. A similar tendency to divide circRNAs in oncogenes and tumor suppressor genes can be now remarked. We presented in this review only circRNAs which are reported by more than one paper. Already two circRNAs, CDRas1 and circHIPK3, were defined to have tumor suppressor potential by some researchers and oncogenic potential by others (44–48, 61–64).

There are hundreds of papers defining circRNAs as cancer biomarkers or tools that can aid the stratification and predict the outcomes of cancer. Already in some cases, the same circRNA was proposed by different groups as a diagnostic tool for multiple types of cancers. We selected for this review only the circRNAs which were linked more than once with neoplastic disease. Twelve different circRNAs fulfill this condition and nine of these are linked to more than one cancer type. Only three circRNAs are, until now, cancer type specific (Table 1). Each of these three specific circRNAs is supported by publications that originate from the same research group and have common authors. The example that best depicts the lack of specificity from our list is cir-ITCH, a circRNA associated with five different types of cancers: ESCC, CRC, lung cancer, HCC, and bladder cancer. This low level of specificity reminds us of the miRNA world, where the same miRNA is “specific” for multiple non-neoplastic and neoplastic diseases. This raises the question of how could these non-specific molecules find their way to the clinic. Two possible interpretations can be given to these observations: (a) circRNAs are a common driving mechanism of oncogenesis or (b) circRNAs are a common byproduct/end-product of oncogenesis. A very important aspect of future research is to determine where circRNAs are localized in the molecular pathogenic pathway.

The discovery of circRNAs in serum/plasma and their abundant presence in blood cells should be treated with caution. CircRNAs could be potential non-invasive biomarkers, and same pitfalls already proven for circulating miRNAs could be also true for circRNAs. Relatively late it has been proven that 58% of serum/plasma circulating miRNAs, which were proposed as cancer-specific biomarkers, are highly expressed in sub-populations of blood cells and only reflect the level of the circulating cells (75). The same can be true with circRNAs, since it was described that circRNAs are abundant in blood cells, predominantly in platelets and erythrocytes (8); hence, the high level of circRNAs from serum/plasma echoing the number of cells. Therefore, a precise characterization of the abundance of circRNAs in different blood cells, a good description of the in serum/plasma transportation mechanism, and the employment of methods that can predict the origin of the circulating circRNAs should be considered as preliminary steps before proposing that circRNA can solve the problem of “liquid biopsy.” Additionally, important steps like the pre-analytical and analytical processes, which can be the cause of supplementary errors and data misinterpretation, should be considered and analyzed before claiming that a circRNA is a circulating cancer specific biomarker.

The majority of the papers providing evidence that circRNAs play a role in cancer offer only limited information about their function. Most of the studies suggest that circRNAs fulfill their function by miRNA sponging. The concept of miRNA sponging became attractive after the competitive endogenous RNA (ceRNA) hypothesis was proposed: ncRNAs interfere with mRNA translation by binding (sponging) miRNAs that were supposed to complementary target the mRNA (76). A number of theoretical papers mathematically modeled this interaction (77, 78) and additional experimental data (79) showed this crosstalk is impossible because of the very low abundance of binding sites harbored by lncRNA or mRNA. The discovery of CDR1as with its repetitive structure and over 70 binding sites for the same miRNA relaunched the hypothesis. However, following *in silico* and experimental studies showed that no other circRNA has this inhibitory potential (25, 28). Therefore, a circRNA that usually is not abundant and has a limited number of interaction sites for a miRNA could not be a potent miRNA sponge and its function remains largely unknown. Moreover, the lack of a clear function of circRNAs reminds us of the fact that only a part of miRNAs are abundant enough in a tissue to truly exert a post-translational regulation of mRNA: miRNAs act more as buffers that maintain the translation in a state of equilibrium (80). Despite this, numerous papers present a dysregulated miRNA as the main mechanism of a pathogenic chain. Therefore, a better characterization of the function of circRNAs is necessary before truly demonstrating their implication in cancer. Moreover, it is important to realize that circRNAs, like miRNAs, are part of a complex molecular network. In order to systematically study their function it is necessary to assess their relationship not only with the linear mRNAs that arise from the common pre-mRNAs, but also the miRNAs and RBPs with which the circular transcript interacts and furthermore their downstream targets.

In conclusion, a basic understanding of the biology (i.e., biogenesis, function, localization, conservation) of circRNAs is necessary before trying to find a clinical application for these new molecules.

## Author Contributions

Conception and design (MD); Provision of study materials (MD, GC); Collection and assembly of data (MD); Manuscript writing: (MD, GC); Figure Design (MD); Final approval of manuscript: (MD, GC).

## Conflict of Interest Statement

The authors declare that the research was conducted in the absence of any commercial or financial relationships that could be construed as a potential conflict of interest.
