# Corrigendum: Circular RNAs in Cancer – Lessons Learned From microRNAs

**DOI:** 10.3389/fonc.2018.00307

**Published:** 2018-09-21

**Authors:** Mihnea Dragomir, George A. Calin

**Affiliations:** ^1^Department of Experimental Therapeutics, The University of Texas MD Anderson Cancer Center, Houston, TX, United States; ^2^Research Center for Functional Genomics, Biomedicine and Translational Medicine, Iuliu Hatieganu University of Medicine and Pharmacy, Cluj-Napoca, Romania; ^3^Department of Surgery, Fundeni Clinical Hospital, Carol Davila University of Medicine and Pharmacy, Bucharest, Romania; ^4^Center for RNA Interference and Non-Coding RNAs, The University of Texas MD Anderson Cancer Center, Houston, TX, United States

**Keywords:** circular RNA, microRNA, non-coding RNAs, cancer, biomarker

In the original article, the details for reference (70) were incorrect. The In Press version of the article was cited instead of the final published article. The full reference appears below. The authors apologize for this error and state that this does not change the scientific conclusions of the article in any way.

The original article has been updated.

## Conflict of interest statement

The authors declare that the research was conducted in the absence of any commercial or financial relationships that could be construed as a potential conflict of interest.
